# Effects of Multibracket Orthodontic Treatment versus Clear Aligners on Periodontal Health: An Integrative Review

**DOI:** 10.3390/dj10100177

**Published:** 2022-09-21

**Authors:** Aaron Jacob David Partouche, Filipe Castro, Ana Sofia Baptista, Liliana Gavinha Costa, Juliana Campos Hasse Fernandes, Gustavo Vicentis de Oliveira Fernandes

**Affiliations:** 1 FP-I3ID, FSC, Universidade Fernando Pessoa, 4249-004 Porto, Portugal; 2 Cooperativa de Ensino Superior, Politécnico e Universitário (CESPU), 4585-116 Gandra, Portugal; 3 Private Office, Ann Arbor, MI 48109, USA; 4 Periodontics and Oral Medicine Department, University of Michigan School of Dentistry, Ann Arbor, MI 48109, USA

**Keywords:** clear aligners, orthodontic treatment, orthodontic multibracket, periodontal health, multibracket appliances, integrative review, multidisciplinary approach

## Abstract

**Objective**: This integrative review aimed to identify studies comparing the periodontal health in patients wearing multibracket orthodontic appliances and clear aligners. **Materials and methods**: An integrative literature search was performed through different databases, PubMed/Medline, PMC, and the Cochrane Library. This work was submitted to a search strategy following the PICO method and included the focus question: “Could the chosen orthodontic appliance change significantly the oral hygiene of the patient, impairing the periodontal health?” This work included analytical and controlled studies on humans published between 2005 and 2020, in the English language, establishing a comparison of the periodontal status in patients undergoing orthodontic multibracket and clear aligners therapies. The main periodontal indexes assessed were plaque index (PI), pocket depth (PD), gingival index (GI), and bleeding on probing (BoP). **Results**: The electronic research displayed 386 articles on PMC, 106 on PubMed, and 40 on the Cochrane Library. After removal, just 25 articles were selected for full-text screening, but just eight studies were eligible for this integrative review. It was enumerated that 204 patients were treated with aligners and 294 with multibracket orthodontic appliances, mainly elastomeric ligated brackets. Only the plaque index displayed a significant difference between the two groups and general data obtained showed a better control for periodontal health in the clear aligners. Limitations such as age, malocclusion severity, therapeutic choice, and different time measure was observed. In addition, the oral hygiene instruction and follow-up by a professional were different, and the role of malocclusion was not present in the studies. **Conclusions**: Within the limitations of this study, better results for periodontal health were found in the clear aligners. Therefore, more studies are necessary to affirm that aligners are synonymous with better gingival conditions in comparison with multibracket appliances. Other variables such as oral hygiene instructions, motivation, and supportive treatment tend to be more prevalent than the type of appliance itself in the periodontal evaluation.

## 1. Introduction

Orthodontic treatment ensures the proper alignment of the teeth and improves the occlusal and jaw relationship. This not only aids in better mastication, speech, and facial esthetics, but also contributes to general and oral health, thereby improving the quality of life [1]. Thus, the demand for orthodontic treatment has increased in both adult and young patients [2,3]. 

Multibracket appliances are the most common and traditional treatment method used in contemporary orthodontics [4]. Conventional orthodontic methods have been associated with a general compromise in facial appearance, raising a major concern among patients seeking orthodontic treatment [5].

To manage esthetic concerns, lingual orthodontics have gained popularity in recent decades, allowing for multibracket mechanics and invisible treatments [6]. Moreover, clear aligner treatment has also been introduced in recent decades to satisfy the esthetic and comfort requirements of adult orthodontic patients. This treatment is based on removable thermoplastic splints covering all the teeth and part of the marginal aspects of the gingiva, which progressively move the teeth into an ideal position [7].

However, similar to any other treatment, orthodontic procedures have complications. Periodontal issues are one of the most observed side-effects associated with orthodontics [8]. In the scientific literature, there is an advantage of clear aligners over multibracket appliances for specifics characteristics, such as in the segmented movement of teeth and shortened treatment duration, whereas braces are more effective in producing adequate occlusal contacts, controlling teeth torque, and retention [9].

Thereby, the goal of this integrative review was to compare the periodontal health of patients undergoing orthodontic treatment with conventional multibracket appliances (brackets) and patients with removable appliances (clear aligners).

## 2. Material and Methods

The problematic of this study was developed through the PICO method (Table 1), with the following focus question: “Could the chosen orthodontic appliance affect significantly the oral hygiene of the patient, impairing the periodontal health?”

The positive hypothesis of this review is that clear aligners are associated to a better periodontal status than multibracket appliances.

### 2.1. Study Design

This work included analytical and controlled studies on humans published between 2005 and 2020, establishing a comparison of the periodontal status of patients undergoing orthodontic treatment with clear aligners and multibracket orthodontic appliances.

Under consideration was Invisalign Technology (Align Technology, Inc., San Jose, CA, USA) for aligners, in general terminology, as well as several types of brackets such as elastomeric ligated brackets, conventional ceramic brackets, and self-ligating brackets used for orthodontic treatment as a common group of multibracket orthodontic appliances (conventional). 

### 2.2. Population

Human; both genders of any age, ethnicity, and malocclusion class; undergoing orthodontic treatment with conventional multibracket appliances or transparent aligners. 

### 2.3. Search Strategy 

An electronic search was undertaken with different combinations of keywords:

(Conventional [All Fields] AND orthodontic [All Fields] AND (“therapy” [Subheading] OR “therapy” [All Fields] OR “treatment” [All Fields] OR “therapeutics” [MeSH Terms] OR “therapeutics” [All Fields])) AND Aligners [All Fields] AND (Periodontal [All Fields] AND (“health” [MeSH Terms] OR “health” [All Fields]))

(orthodontic appliances, braces” [MeSH Terms] OR (“orthodontic” [All Fields] AND “appliances” [All Fields] AND “multibracket” [All Fields]) OR “multibracket orthodontic appliances” [All Fields] OR (“multibracket” [All Fields] AND “appliances” [All Fields]) OR “multibracket appliances” [All Fields]) AND Aligners [All Fields] AND (Orthodontic [All Fields] AND (“therapy” [Subheading] OR “therapy” [All Fields] OR “treatment” [All Fields] OR “therapeutics” [MeSH Terms] OR “therapeutics” [All Fields])) AND (periodontal [All Fields] AND (“health” [MeSH Terms] OR “health” [All Fields]))

Aligners [All Fields] AND (Orthodontic [All Fields] AND (“therapy” [Subheading] OR “therapy” [All Fields] OR “treatment” [All Fields] OR “therapeutics” [MeSH Terms] OR “therapeutics” [All Fields])) AND (Periodontal [All Fields] AND (“health” [MeSH Terms] OR “health” [All Fields]))

### 2.4. Study Selection and Eligibility Process 

A comprehensive electronic search was conducted in February 2020 to identify relevant publications to build this work. PubMed/Medline, PMC, and Cochrane Library databases were used. The search was performed by the author (A.J.D.P.), assisted and supported by another author (F.C.).

MeSH terms to target relevant orthodontic studies were used. Only English language restrictions were applied. The bibliographies of the included studies were also used to identify cross-additional studies for possible inclusion.

**Figure 1:**
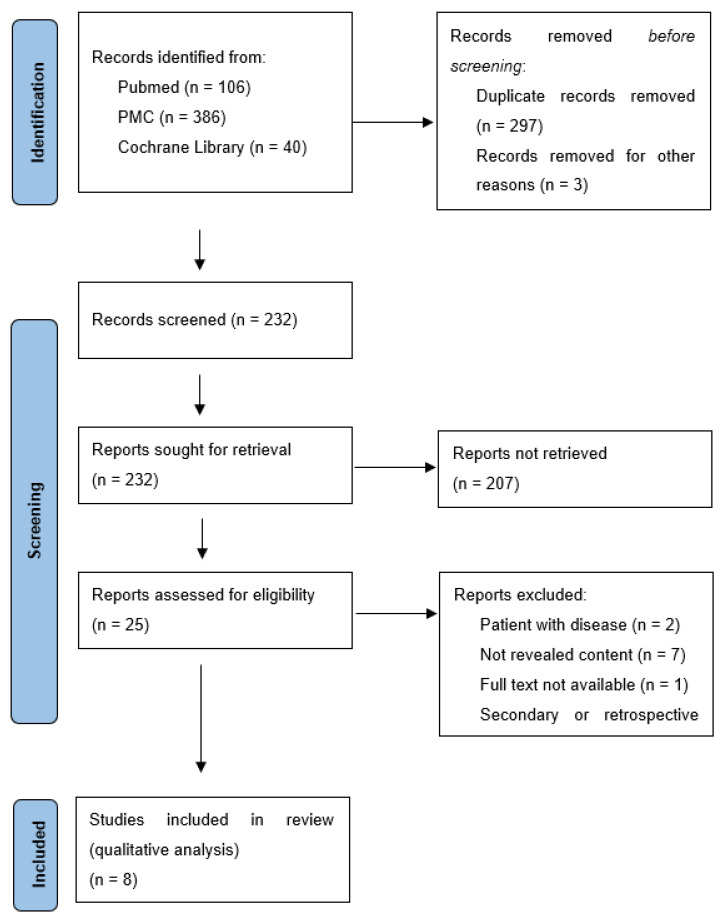
Research PRISMA Flow diagram.

The selection and eligibility process are illustrated in the PRISMA Flow Diagram (Figure 1). The authors established the criteria. Then, the studies were initially screened by title and abstract, in accordance with the inclusion/exclusion criteria (Table 2). Following this, the studies were reviewed at the full-text level and agreement was obtained at the two stages, and if needed, a third author was consulted (G.V.O.F.).

### 2.5. Data Items 

One customized data abstraction form was used to extract data from each study. The following variables were recorded: authors (references) and the year of the study, gender and quantity, average age, recruitment time, study design, country referred to in the study, outcome measure involved, follow-up in months, sample sizes in relation to the appliance used, and control groups.

## 3. Results 

### 3.1. Search Results

The electronic research displayed 386 articles on PMC, 106 on PubMed, and 40 on the Cochrane Library. After duplicates were removed and titles and abstracts revised, 25 articles were selected for full-text screening. To finish, analyzing the full text and according to the inclusion-exclusion criteria, seven secondary studies were excluded, two studies involving patients with periodontal disease, seven articles without interest for this work, and only one study due to the unavailability of the full text. At the end, eight studies were approved for this integrative review (Figure 1).

### 3.2. Introduction of the Selected Studies

This shortcoming did not allow us to retrieve meta-analysis data from the included papers (*n* = 8). Seven analytical studies (four prospective cohort studies, two cross sectional studies, and one randomized controlled study) were enumerated. Female gender was dominant. Miethke & Brauner [10] included a control group of 30 patients wearing aligners from another investigation, which is also included in this work.

Several brackets were used, such as elastomeric-ligated brackets and conventional ceramic brackets; Chhibber et al. [11] and Issa et al. [12] considered self-ligated brackets; only Miethke & Brauner [10] used multibracket lingual appliances. Moreover, different follow-up and outcome measures were used. Only Abbate et al. [13] and Chhibber et al. [11] submitted patients to long-term follow-up examination. The characteristics of the eligible studies are regrouped in Table 3. The gingival index (GI), pocket depth (PD), plaque index (PI), and bleeding of probing (BoP) were evaluated and reported in Table 4.

### 3.3. Indexes Comparison

The eight studies presented different tools for the comparative evaluation of patients’ periodontium and time for assessment. Only two studies, Miethke & Vogt’s [14] and Miethke & Brauner’s [10], presented the same indexes: respectively, GI, PBI, PI, SPD, and the same time measure, which corresponded to three different periods with 21/28 days of interval.

Following the idea of comparing the highest quantity of data available among the different studies, different outcome measures were selected according to their frequency. In this sense, the PI used in all the studies was selected, as well as PD (6/8), GI (6/8), and BoP (6/8) for the quantitative analysis.

The methods employed to realize index evaluations were not the same among the studies. GI, PD, and BoP described no difference between patients undergoing orthodontic treatment with multibracket appliances and with clear aligners, even if the global outcome described a lower value in favor of aligners. The PBI index, not mentioned in Table 4, also showed no significative difference between both appliances. Only the plaque index was associated t a significative difference. A large spectrum of time treatment was used relative to the eight studies selected, which made it difficult to be evaluated. Between the period of 3 and 9 months, most indexes saw an increase in both appliances, especially in multibracket orthodontic appliances. 

### 3.4. Studies’ Details

Pango Madariaga et al. [15] demonstrated that only BoP significantly increased in multibracket appliances, as compared with aligners at the baseline evaluation (respectively, 0.77 versus 0.55; *p* = 0.006). Those results decreased and became similar at T3 (0.13 for both appliance). An intra group comparison showed statistically significant decreases between T0 and T3 in both groups for PD, BoP, and PI. This study introduced a new index (REC) describing higher value in the aligners group. Nevertheless, the authors concluded that the type of appliance did not have any effects on the improvement in periodontal variables, neither aging nor number of sites evaluated, even if they were significant, giving more credit to other criteria.

In the same courant, Chhibber et al. [11] also contradicted population beliefs that removable appliances compare to multibracket ones are less subject to undesirable effects on periodontal health. If the aligners group described the lowest mean values for PI, GI, and PBI in comparison with self-ligated and conventional elastomeric brackets, the odds ratios at T18 were not significant, pointing out no evidence of differences in terms of the level of oral hygiene for the three types of appliances. This is also true for the PI index; none of the odd ratios were significantly different from 1 (*p* > 0.05) at the three-time evaluation. In addition, the results between SLB and ELB were almost similar. However, the odds ratios comparing aligners and conventional brackets for GI (OR = 0.14; *p* = 0.015) and PBI (OR = 0.10; *p* = 0.012) were statistically significant at T9, suggesting that aligners performed better for a short time (these indexes were more than twice as high for FG between T0 and T9 and almost stable for aligners). They should be 86% less likely than the multibracket group to have a degree of periodontal inflammation and 90% less likely to have papillary bleeding, which the authors concluded the choice of orthodontic appliance has little impact on the clinical periodontal parameters.

Levrini et al. [16] described a statistically significant difference between both groups for PI, BoP, and PD, with the aligner’s patients being associated to the lowest mean values. In this prospective study, the intra-group comparison showed the worst periodontal parameters scores regardless of the indexes, increasing at T3 in the multibracket group, as well as the total biofilm mass. Otherwise, statistics were not mentioned. Aligners showed a statistically significant increase only for PI at T3, but the results were not present. A real-time PCR analysis revealed a statistically significant difference in the total biofilm mass with a lower score in the 90 days follow-up examination for the aligners group. Moreover, the microbiological analyses detected the presence of *A. actinomycetemcomitans* in one patient subject to multibracket appliances at T1 and T3. In this track, the mean bacterial concentration “C” was also significantly lower in aligners, corroborating a bigger plaque accumulation in the multibracket group. The authors concluded that removable appliances must be considered as a first treatment option in patients subject to periodontal disease.

Following the same dynamic, Abbate et al. [13] also conducted a microbiological analysis; however, none of the patients tested were subject to any periodontopathic anaerobes after 12 months of treatment. From the baseline until 1 year of treatment, the full mouth plaque (FMPS) value tripled, and full mouth bleeding (FMBS) doubled for teenagers treated with multibracket appliances; both scores were reduced in the teenagers wearing aligners. According to PI and BoP, it can underline a completely significant opposite trend in time between the two groups: both indexes progressively increased for the multibracket appliances (PI from 0.82 to 2.42), contrasting with a continuous decrease in patients using aligners (PI from 0.91 to 0.36), suggesting less plaque accumulation and gingival inflammation in this case. Moreover, the sulcus probing increased in all the treated patients, especially in the multibracket group (from 2.26 to 3.42), while it remained stable in the aligners group (from 2.28 to 2.5). The authors assessed the patients’ compliance with the oral hygiene, displaying a favorable significative difference for removable treatment.

Azaripour et al. [17], through the API/MPI index, described an increase in dental plaque in both appliances, which was higher in the multibracket group (37.7 ± 21.9%) in comparison with aligners (27.8 ± 24.6%). Nevertheless, the difference was not significant at T12. The authors displayed significantly lower gingival inflammation for aligners patients (cf. Chhibber et al. [11]). Indeed, if GI and SBI increased in the aligners group, those indexes were almost doubled between the initial and ending time of treatment (T12) for the multibracket group (GI: from 0.29 ± 0.24 to 0.54 ± 0.50 and SBI: from 7.2 ± 4.4 to 15.2 ± 7.6). 

In Miethke & Vogt’s [14] clinical trial, all the indexes described a basic improvement from the first to the third screening, regardless of the orthodontic appliance. Initially, no statistically significant differences were observed for GI, PBI, and SPD. The one exception was PI, which was already significantly different at the first evaluation (multibracket group on average 0.32 > than aligners). Moreover, the scores comparison from all three evaluation time points showed a significantly lower PI for patients treated with aligners. The most superficial improvement concerns SPD. In other terms, the authors concluded that there were no differences initially and during treatment between multibracket appliances and aligners, crediting the improvement of oral hygiene with other criteria.

In a second study, Miethkhe & Brauner [10] established the same work but bonded the brackets on the lingual/palatine surface. The SPD was slightly increased in both study groups but did not differ significantly; minor changes might be associated to superficial periodontal disease, according to the authors. Unsurprisingly, the GI and PI scores at the first screening were twice as high, and the PBI half as high, in patients wearing brackets. All three indexes were significantly worse at the second and third screening, standing in stark contrast with the aligners group and showing how complex oral hygiene is in this case.

Issa et al. [12] also demonstrated the difference in terms of plaque levels, which were much lower in aligners patients than those undergoing conventional treatment. Moreover, patients treated with aligners showed better scores in all of the seven indexes recorded: PI, GI, GBI, SBI, PBI, BPE, and BoP. Only BoP showed no significant differences (*p* = 0.704). This result might be explained by patient compliance with oral hygiene instructions. In this study [12], the authors mentioned the Basic Periodontal Examination index in order to evaluate the periodontal heath. Moreover, the results revealed no significant differences between self-ligating brackets and aligners, suggesting a better control of oral hygiene with this type of bracket over conventional or ceramic brackets.

## 4. Discussion

Periodontal health is an important factor that may be used to evaluate the success of orthodontic therapy. Periodontal complications are reported to be one of the most common side effects linked to orthodontics. The periodontal complications associated with orthodontic therapy are mainly gingivitis, periodontitis, and gingival recession [1]. However, the risk and complication associated with treatment are reported to be considerably lower compared to other surgical or nonsurgical interventions [18].

The presence of microbial plaque is reported to be the most important factor in the initiation, progression, and recurrence of periodontal disease [19]. If results, in terms of significance, are contrasted among authors, it is clearly established that multibracket orthodontic appliances can retain more dental plaque, a vector of gingival inflammation. Indeed, orthodontics brackets and elastics might interfere with the effective removal of dental plaque, thereby increasing the risk of gingivitis.

A few clinical studies also reported poor periodontal health and greater loss of clinical attachment level distally in the dental arches. This could be a result of poor oral hygiene in the molar regions and the presence of molar bands, which favors food lodgment [20]. The gingival, distal, and mesial areas, in relation to the brackets, attracted more biofilm than the occlusal areas, which was mostly due to the interference of arch wires and ligating devices on tooth brushing. There is also relatively less self-cleaning from natural chewing in these areas [21].

The presence of multibracket orthodontic appliances encourages the growth and retention of dental plaque, which results in localized gingivitis [22]. The problem of the lack of adequate microbial plaque removal is greater when undergoing orthodontic treatment [23,24]. Plaque accumulation can favor the transition of the microbial biofilm to a more aggressive periodontopathogen flora in sub-gingival periodontal pockets and the production of pro-inflammatory cytokines [25].

Abbate et al. [12] and Levrini et al. [16] investigated staining, periodontal health, and total biofilm mass through microbiological analysis (3 months follow-up), with patients wearing multibracket orthodontic appliances, and promoted that clear aligners may be a first treatment option in patients with a risk of periodontitis. Furthermore Levrini et al. [16] displayed only one patient undergoing multibracket orthodontic treatment subject to *A. actinomycetemcomitans.* In this sense, the prospective study of Ristic et al. [26] concluded that multibracket appliances in adolescents may transitionally increase the values of all periodontal indexes, pointing out a maximum value at 3 months of treatment and stimulating the growth of periodontopathogen bacteria, but without destructive effects on deep periodontal tissues.

Mummolo et al. [27] displayed a different trend in the bacterial colonization of *S. mutans* and Lactobacilli, and the plaque index in both appliances. The maintenance of a better macroscopic (PI) and microscopic (*S. mutans* and *Lactobacilli*) oral hygiene level in patients with removable appliances should be related to the absence of multibracket retentive surfaces on the patient’s teeth and with the consequent facilitation of oral hygiene procedures. This conclusion is consistent with Levrini et al.’s work [16]. Increased levels of *S. mutans* and *lactobacillus* species have also been reported to be detected in the oral cavity after bonding orthodontic attachments, and some studies have reported that there is a positive correlation between dental caries and the degree of infection with these bacterial species [28].

In this integrative review, different indexes were evaluated: GI, PD, PI, BoP, GBI, SBI, PBI, API/MPI, REC, and BPE. The lack of consensus between studies and authors was obvious. Nevertheless, most of them agreed that only PI showed a significant difference/improvement in patients treated with clear aligners in comparison with those treated with multibracket orthodontic appliances. In Miethke and Vogt [14], Miethke and Brauner [10], and Abbate et al. [13] the plaque index decreased with time, while Issa et al. [12] displayed a significant difference between both appliances. Several explanations were plausible. On this hand, wearing a traditional brace will make people feel uncomfortable, and it is difficult to clean through conventional methods. Patients must carefully brush each bracket and gloss around the wires to remove all traces of plaque, in order to reduce the risk of demineralization during this treatment [29]. This is especially true for multibracket lingual appliances, in which the frequent plaque deposition is not surprising, as almost 60% of all patients wearing lingual appliances complain about significant difficulty with tooth brushing [30].

On the other hand, unlike multibracket dental appliances, removable orthodontic appliances, can be taken out and, thus, enable patients to practice oral hygiene procedures under ideal conditions [31]. Another possibility is that aligners cover the majority of the crown, preventing the accumulation of dental plaque on the teeth, as well as the transition of supragingival dental plaque to subgingival tissues, undeniably leading to potential destruction. A positive relationship between removing the appliance before eating/drinking and compliance with oral hygiene, turning those patients more sensitive to oral care, might be mentioned. Moreover, aligners are more prevalent in the adult population in which oral hygiene education is less complex than adolescents. They are also more cooperative in following the instruction of orthodontists [32]. Considering this, Abbate et al.’s [13] work showed a significant difference in compliance with oral hygiene between patients with aligners and those with a multibracket orthodontic appliance. 

For Miethke and Vogt [14], Abbate et al. [13], Azaripour et al. [17], Levrini et al. [16], and Issa et al. [12] the patients treated with clear aligners have a better periodontal health than those treated with multibracket appliances. For Miethke & Vogt [14], Chhibber et al. [11], and Pango Madariaga et al. [15], the results contradicted this affirmation and accredited other explanations. The almost day-long coverage of all tooth surfaces increases the accumulation of soft matter, which in turn could lead to sub-chronic inflammation. Further, the margins of aligners, almost never perfectly smooth, can irritate the marginal gingivae [14]. Other authors [33] reported an interference with the flushing effect of saliva on dental tissues due to permanent coverage of the surface teeth. Moreover, insufficient saliva secretion reduces the self-cleansing mechanisms of the oral cavity and limits the antimicrobial effects of the residual saliva, which can lead to a greater accumulation of dental plaque [34,35]. Only Chhibber et al. [11] suggested that aligners may offer superior gingival conditions in the short-term of treatment; however, all of them highlighted the crucial role of oral hygiene procedure, which was more important than the type of appliance chosen.

In this sense, Miethke & Vogt [14] and Madariaga et al. [15] raised no evidence of differences between both appliances when regular check-ups and oral hygiene instructions are performed. Chibber et al.’s work [11] extended this conclusion to long-term treatment. To the authors, great importance should be placed on the motivation of the patient, including regular check-ups and by personalizing home-hygiene technique. Other studies [36,37] reinforced this conclusion.

Moreover, two important issues are the treatment duration and case complexity [9]. It is obvious that difficult cases cannot be treated simply or only with clear aligners. Therefore, the choice becomes compulsory. In addition, only skilled orthodontists can achieve good results by using clear aligners.

### Limitations 

Orthodontic treatment time refers to several variables such as age, malocclusion severity, therapeutic choice, etc. Among the eight studies included, different time measures were considered. Only one study focused on long-term evaluation, and one study did not specifically mention the period of evaluation. The results revealed that all the indexes increased at 6 months, but most of the studies reached their last evaluation after 3 months.

Oral hygiene instruction and follow-up by a professional were different: in some studies, the patients were naive about oral hygiene instruction, but in others they received instructions and prophylactic treatment before initiating the orthodontic treatment, which undeniably influenced the results.

The role of malocclusion in periodontal health is important [2] but was not present in the studies. Periodontal parameters were assessed differently according to the studies: technique (Ramfjord system, etc.) and material were not the same, considering the probe, number of teeth, and number of surfaces involved. Some authors did not included their evaluation method. Moreover, methods for measuring the indexes were not similar (FMPS, API, etc.). The ages between patients differed, with some studies considering adults and other adolescents, revealing heterogeneity. Another limitation was the lack of meta-analysis. The studies included in this work were mainly prospective cohort studies with only one randomized control study. This led to an insufficient amount of information for comparison, and bias may appear.

## 5. Conclusions 

Within the limitations of this study, patients treated with clear aligners are less subject to plaque accumulation than those with multibracket orthodontic appliances. However, among all the indexes evaluated, only PI differed significantly. The placement of the multibracket appliances might impact the oral microflora in the short term. Instructions and oral care are important actors during orthodontic treatment and influenced periodontal results, which were heterogenous among the studies. More studies, especially randomized controlled trials, are needed to fully demonstrate that aligners offer better periodontal conditions.

## Figures and Tables

**Table 1:** Description of the search strategy under PICO method.

Population (P)	Patients under Orthodontic Treatment with Multibracket Orthodontic Appliances or Clear Aligners
**Intervention (I)**	Patients treated with clear aligners
**Comparison (C)**	Patients using multibracket orthodontic appliances
**Outcome (O)**	Periodontal health

**Table 2:** Eligibility criteria.

Inclusion Criteria	Exclusion Criteria
Clinical study on human fitting with the subject	Narrative review
RCT, prospective or cohort study comparing periodontal indexes of patients treated with multibracket appliance and aligners with follow-up	Retrospective study
RCT, prospective or cohort study comparing periodontal indexes of patients treated with multibracket appliance and aligners without follow-up	Secondary study
	Articles without clinical studies
Articles in English	No full-text available
Studies fitting with the subject using Invisalign technology	No relevant title or abstract
	Patient with antibiotic therapy or periodontitis

**Table 3:** Characteristics of the eligible studies.

Authors (Refs.)	Female/Male	Sample Number	Average Age	Recruitment Time	Study Design	City/Country	Outcome Measures	Time Measures	Number of Multibracket Appliances	Number of Clear Aligners	Potential Biases
Pango Madaraiga et al., 2020	20/20	Total: 40	Mean age FG: 20.6 Mean age CA: 34.7	Unknown	Prospective clinical study	Naples, Italy	PD PI BoP REC	T0 T3	20	20	Without long-term assessment
Chhibber et al., 2017	30/41	Total: 71	Mean age 15.6 ± 4.3	2011–2014	Randomized control trial	Connecticut, Australia	PI GI PerioBl	T0 T9 T18	44 22 ELB 22 SLB	27	Different periods of assessment (without short-term)
Levrini et al., 2015	52/25	Total 77 (33: Control)	16 to 30 years old Mean age: 24.3	Unknown	Prospective study	Varese, Italy	PI PD BoP Microbiological analysis	T0 T1 T3	35	32	Without long-term of assessment
Abbate et al., 2015	Unknown	Total: 50	10 to 18 years old	2012–2013	Prospective study	Varese, Italy	PD PI BoP Microbiological analysis	T0 T3 T6 T12	25	25	Does not specify female/male ratio; different periods of assessment
Azaripour et al., 2015	73/27	Total: 100	11 to 62 years old Mean age FG: 16.3 Mean age CA: 31.9	Unknown	Cross-sectional study	Gutenberg, Germany	GI SBI API MPI	T0 T12	50	50	Different periods of assessment and indexes; without short-term assessment; included children
Miethke & Vogt, 2005	43/17	Total: 60	18 to 51 years old Mean age: 30.1	2002–2003	Clinical trial (Prospective cohort study)	Berlin, Germany	GI PBI PI SPD	T1 T2 T3 3/4 weeks intervals	30	30	Without long-term assessment
Miethke & Brauner, 2007	Unknown	Total: 60	16 to 48 years old Mean age: 39.6	Feb and May of 2005	Prospective study	Berlin, Germany	GI PBI PI SPD	T1 T2 T3 3/4 weeks intervals	30 (lingual group)	30 (control group from the previous study)	Does not specify female/male ratio; without long-term assessment
Issa et al., 2020	40/40	Total:80	Mean age CA: 26,85 Mean age FG: 27,05	2015–2016	Cross-sectional study	China	PI GI GBI SBI PBI BPE BoP	regular assessments unknown	60 20 ELB 20 CCB 20 SLB	20	No deep information about time of assessment

**Table 4:** Indexes evaluation.

Authors (Refs.)	Gingival Index (GI)			Probing Depth (PD)			Plaque Index (PI)			Bleeding of Probing (BoP)		
Pango Madaraiga et al., 2020	not mentioned	not mentioned	not mentioned	AG T0 = 0.1 T3 = 0	FG T0 = 0.3 T3 = 0.14	Mean ∑AG = 0.05 ∑FG = 0.22	AG T0 = 0.42 T3 = 0.11	FG T0 = 0.31 T3 = 0.15	Mean ∑AG = 0.27 ∑FG = 0.23	AG T0 = 0.55 T3 = 0.13	FG T0 = 0.77 T3 = 0.13	Mean ∑AG = 0.34 ∑FG = 0.9
Chhibber et al., 2017	AG T0 = 0.42 ± 0.5 T9 = 0.50 ± 0.59 T18 = 0.75 ± 0.53	FG T0 = 0.05 ± 0.22 T9 = 1.21 ± 0.79 T18 = 1.32 ± 0.67	Mean ∑AG = 0.55 ± 0.54 ∑FG = 1.01 ± 0.56	not mentioned	not mentioned	not mentioned	AG T0 = 0.50 ± 0.51 T9 = 0.83 ± 0.48 T18 = 0.92 ± 0.58	FG T0 = 0.70 ± 0.73 T9 = 1.32 ± 0.67 T18 = 1.32 ± 0.67	Mean ∑AG = 0.75 ± 0.52 ∑FG = 1.1 ± 0.69	not mentioned	not mentioned	not mentioned
Levrini et al., 2015	not mentioned	not mentioned	not mentioned	AG T0 = 2.18 T1 = 2.75 T3 = 1.6	FG T0 = 2.18 T1 = 2.2 T3 = 1.3	Mean ∑AG = 2.17 ∑FG = 1.89	not mentioned	not mentioned	not mentioned	not mentioned	not mentioned	not mentioned
Abbate et al., 2015	not mentioned	not mentioned	not mentioned	AG T0 = 2.28 T3 = 2.23 T6 = 2.37 T12 = 2.5	FG T0 = 2.26 T3 = 2.86 T6 = 3.22 T12 = 3.42	Mean ∑AG = 2.35 ∑FG = 2.94	AG T0 = 0.91 T3 = 0.64 T6 = 0.32 T12 = 0.36	FG T0 = 0.82 T3 = 1.92 T6 = 2.32 T12 = 2.42	Mean ∑AG = 0.56 ∑FG = 1.87	AG T0 = 0.14 T3 = 0 T6 = 0.04 T12 = 0.04	FG T0 = 0 T3 = 0.36 T6 = 0.58 T12 = 0.74	Mean ∑AG = 0.05 ∑FG = 0.42
Azaripour et al., 2015	AG T0 = 0.27 ± 0.25 T12 = 0.35 ± 0.34	FG T0 = 0.29 ± 0.24 T12 = 0.54 ± 0.50	Mean ∑AG = 0.31 ± 0.29 ∑FG = 0.42 ± 0.37	not mentioned	not mentioned	not mentioned	AG T0 = 0.16 ± 0.1 T12 = 0.30 ± 0.25	FG T0 = 0.20 ± 0.1 T12 = 0.38 ± 0.22	Mean ∑AG = 0.23 ± 0.18 ∑FG = 0.58 ± 0.16	not mentioned	not mentioned	not mentioned
Miethke & Vogt, 2005	AG T1 = 0.71 ± 0.39 T2 = 0.61 ± 0.35 T3 = 0.46 ± 0.34	FG T1 = 1.02 ± 0.69 T2 = 0.73 ± 0.58 T3 = 0.68 ± 0.66	Mean ∑AG = 0.59 ± 0.30 ∑FG = 0.81 ± 0.59	AG T1 = 2.39 ± 0.45 T2 = 2.29 ± 0.41 T3 = 2.26 ± 0.48	FG T1 = 2.60 ± 0.73 T2 = 2.52 ± 0.65 T3 = 2.50 ± 0.67	Mean ∑AG = 2.31 ± 0.39 ∑FG = 2.45 ± 0.65	AG T1 = 0.48 ± 0.41 T2 = 0.41 ± 0.37 T3 = 0.28 ± 0.32	FG T1 = 0.80 ± 0.58 T2 = 0.56 ± 0.44 T3 = 0.50 ± 0.53	Mean ∑AG = 0.39 ± 0.31 ∑FG = 0.62 ± 0.48	not mentioned	not mentioned	not mentioned
Miethke & Brauner, 2007	AG T1 = 0.71 ± 0.39 T2 = 0.61 ± 0.35 T3 = 0.46 ± 0.34	FG T1 = 1.02 ± 0.53 T2 = 1.02 ± 0.43 T3 = 0.96 ± 0.43	Mean ∑AG = 0.59 ± 0.30 ∑FG = 1.00 ± 0.43	AG T1 = 2.39 ± 0.45 T2 = 2.29 ± 0.41 T3 = 2.26 ± 0.48	FG T1 = 2.55 ± 0.38 T2 = 2.43 ± 0.33 T3 = 2.50 ± 0.35	Mean ∑AG = 2.31 ± 0.39 ∑FG = 2.50 ± 0.33	AG T1 = 0.48 ± 0.41 T2 = 0.41 ± 0.37 T3 = 0.28 ± 0.32	FG T1 = 0.84 ± 0.46 T2 = 0.95 ± 0.44 T3 = 0.89 ± 0.45	Mean ∑AG = 0.39 ± 0.31 ∑FG = 0.89 ± 0.41	not mentioned	not mentioned	not mentioned
Issa et al., 2020	AG Tx = 0.008	FG Tx = 1.06	—	not mentioned	not mentioned	not mentioned	AG Tx = 0.2	FG Tx = 1.7	—	AG Tx = 0.01	FG Tx = 0.37	—

AG = aligners group; FG = multibracket appliance group; gingival index (GI); pocket depth (PD); plaque index (PI); and bleeding of probing (BoP).

## Abbreviations

AG	Aligners group
API	Approximative plaque index
BoP	Bleeding of probing
CCM	Conventional ceramic brackets
ELB	Elastomeric-ligated brackets
FG	Fixed group
FMBS	Full mouth bleeding score
FMPS	Full mouth plaque score
GI	Gingival index
MPI	Modified plaque index
PBE	Periodontal basic examination
PBI	Papillary bleeding index
PD	Probing depth
PI	Plaque index
REC	Gingival recession
SBI	Sulcus bleeding index
SLB	Self-ligated brackets
Tn	Time measure in months
